# Secukinumab (Anti‐IL‐17A Therapeutic Antibody) Improves Clinical Outcome for a Mixed Endotype CRS

**DOI:** 10.1002/ccr3.9692

**Published:** 2024-12-09

**Authors:** Mihkel Plaas, Agnieska Brazovskaja, Kai Kisand, Liina Tserel, Pärt Peterson

**Affiliations:** ^1^ Ear Clinic Tartu University Hospital Tartu Estonia; ^2^ Institute of Clinical Medicine University of Tartu Tartu Estonia; ^3^ Institute of Biomedicine and Translational Medicine University of Tartu Tartu Estonia

**Keywords:** biological treatment, biologics, chronic rhinosinusitis with polyps, CRSwNP, dupilumab, secukinumab

## Abstract

We identified two CRSwNP patients who had previously failed treatment with an anti‐IL‐4/IL‐13 antibody (dupilumab). Based on their clinical characteristics and blood cytokine levels, we considered them mixed Type II/Type III cases and treated them with an anti‐IL‐17 antibody (secukinumab). Anti‐IL‐17 antibody secukinumab was superior in reducing their NPS and SNOT‐22 values compared to dupilumab. IL‐17 could be a promising target for non‐type II and mixed endotype CRS treatment.

## Abstract 2

In this case report we describe two patients with a mixed Type II/Type III endotype CRSwNP who, after failing treatment with dupilumab, showed favourable treatment outcomes with secukinumab (anti‐IL‐17A antibody) treatment.

## 1 Introduction

Primary chronic rhinosinusitis (CRS), a chronic inflammation of the nose and paranasal sinuses, is driven either by CD4^+^ Th1 (Type I), Th2 (Type II), or Th17 (Type III) cell‐regulated inflammation. Type I and type III are often collectively referred to as non‐type II. Clinically, CRS presents either with (CRSwNP) or without (CRSsNP) polyps. While most cases of CRSwNP can be attributed to type II inflammation, a considerable proportion are either non‐type II or mixed endotype cases (presenting with signs and symptoms characteristic of more than one endotype). However, therapeutic antibodies currently indicated for difficult‐to‐treat CRSwNP cases (e.g., dupilumab, mepolizumab) all target type II inflammation. At present there is an unmet need for difficult‐to‐treat CRSwNP patients with either non‐type II or mixed endotype [1, 2].

Currently, CRS endotyping is primarily based on phenotypic characteristics (e.g., comorbid asthma, need for steroid therapy) and surrogate markers like elevated IgE levels and eosinophilia [1]. However, these are unspecific and do not conclusively identify CRS endotypes [3]. Failure to definitely distinguish different endotypes could at least partially explain some cases of unsatisfactory treatment response to conventional monoclonal antibody therapy. In this limited case series, we report two CRSwNP patients who previously had failed treatment with an anti‐IL‐4/IL‐13 antibody (dupilumab) but showed favorable outcome with an anti‐IL‐17A antibody (secukinumab). The study was approved by the ethics committee of Estonian State Agency of Medicines and was conducted in accordance with the Good Clinical Practice Guideline. Informed consent was obtained from all participants.

## 2 Case History

### 2.1 Case 1

Subject S1 (see Table 1) was a 25 year old male with a history of CRSwNP (with multiple surgical interventions), asthma, and allergy. He initially presented with high blood eosinophile count (640 × 10^6^), elevated IgE (194 kU/L), and anosmia and was thus considered as type II CRSwNP. He was initially included in a small‐scale clinical study with a total of 13 participants to evaluate the treatment effects of an anti‐IL‐4/IL‐13 antibody (dupilumab) but showed minimal‐ to no treatment response over the 10 week treatment period (NPS 6 ➔ 5, see Table 2).

**TABLE 1:** Patient characteristics.

	S1	S2
Age	25	33
Sex	Male	Female
Asthma	Yes	Yes
Allergy	Yes	No
(Previous) need for steroids	Yes	Yes
Endoscopic sinus surgeries	3	1
NSAID intolerance	Yes	Yes
Hyposmia/anosmia (subjective)	Anosmia	Hyposmia
Blood eosniphile count	640 × 10^6^	640 × 10^6^
Blood IgE count	194 kU/L	71.4 kU/L

**TABLE 2:** Treatment response for S1.

	Dupilumab pre‐treatment	Dupilumab post‐treatment^a^	Secukinumab pre‐treatment	Secukinumab post‐treatment^a^
SNOT‐22	73	68	63	24
NPS	6	5	5	3

^a^ Final timepoint was 12 weeks after the first dose for dupilumab and 16 weeks for secukinumab.

### 2.2 Case 2

S2 (Table 1) was a 33 year old female with CRSwNP and asthma but no previous history of endoscopic sinus surgery. She presented with nasal polyps, asthma, hyposmia, and elevated blood eosinophile count (640 × 10^6^). Based on her phenotype, she was also initially categorized as type II endotype and as a part of a clinical study received dupilumab for 10 weeks. Similarly to S1 her treatment response was poor with no reduction in her NPS values (see Table 3).

**TABLE 3:** Treatment response for S2.

	Dupilumab pre‐treatment	Dupilumab post‐treatment^a^	Secukinumab pre‐treatment	Secukinumab post‐treatment^a^
SNOT‐22	23	8	30	19
NPS	5	5	5	1

^a^ Final time point was 12 weeks after the first dose for dupilumab and 8 weeks for secukinumab for S2. By week 16 (8 weeks after the last dose) her NPS score had returned to pre‐preatment levels (1➔5).

In summary, both subjects showed minimal‐to‐no treatment response to dupilumab and in addition presented with signs of type II (anosmia, polyps, blood eosinophilia) and type III endotype (highest relative IL‐17 values). We therefore hypothesized that both subjects may belong to a mixed Th2/Th17 endotype and could benefit from anti‐IL‐17 treatment.

## 3 Methods

During the initial study each participant (including subjects S1 and S2) received 300 mg of dupilumab bi‐weekly (six doses in total) for 10 weeks. We analyzed blood‐based biomarkers prior to the treatment period using the high‐throughput proteomic platform “Olink Target 96 Inflammation.” Given that we used a semi‐quantitative detection method, we could only compare inter‐group differences; therefore, biomarker data are reported as relative rather than absolute values. Clinical treatment response was measured using the sino‐nasal outcome test (SNOT‐22) and nasal polyp score (NPS).

As part of a follow‐up study, S1 and S2 received secukinumab treatment for 12 weeks (one 150 mg injection on weeks 0, 1, 2, 3, 4, 8, and 12). Since secukinumab has never been evaluated in the context of CRS we used the dosing scheme approved for psoriasis treatment by the drug manufacturer. Of note, both subjects were using the same base‐treatment (mometasone nasal spray) during both biological treatment courses.

Final timepoints to record clinical data were chosen based on recommended minimal gaps between the injections; 2 weeks after the last dose for dupilumab (week 12) and 4 weeks after the last dose for secukinumab (week 16).

## 4 Results

No serious adverse effects were noted during either treatment course. No rescue medicine (oral steroid therapy) or rescue surgery was needed during the study periods but both subjects underwent endoscopic sinus surgery after treatment with dupilumab with very short remission times and fast regrowth of polyps illustrated by their NPS values prior to secukinumab treatment.

Blood cytokine profile analysis measured before the first dupilumab dosing revealed that S1 and S2 had the highest relative serum IL‐17A levels (S1 = 183% and S2 = 173% of the cohort average levels) of the cohort and relatively low levels of IL‐4 (S1 = 64% and S2 = 109% of the cohort average). Th2 cytokines IL‐5 and IL‐13 serum levels were below detection threshold.

A direct comparison of treatment arms (Tables 2 and 3) showed that greater NPS score reduction was achieved with secukinumab (S1 = 2 points, S2 = 4 points) versus dupilumab (S1 = 1 point, S2 = 0 points). For S1, secukinumab was also superior in reducing the SNOT‐22 score (39 points) versus dupilumab (5 points). S2 had low baseline SNOT‐22 values prior to both biological treatment courses and no meaningful difference was noted between the treatment arms. S2 opted not continue with secukinumab after week 4 (after the 5th dose) for personal reasons but agreed to return for pre‐arranged follow‐up visits. At 16‐week follow‐up her NPS score had returned to pre‐treatment levels. The data that support the findings of this study are available from the corresponding author (MP), upon request.

## 5 Discussion

The pro‐inflammatory cytokine IL‐17 is a key regulator of type III inflammation and in addition to CRS has been implicated in several diseases characterized by chronic inflammation, including psoriasis, rheumatoid arthritis, and asthma [4]. For instance, the IL‐17 blocking antibody secukinumab is indicated for psoriasis treatment. Interestingly, IL‐17 has also emerged as a potential therapeutic target for CRS in preclinical animal models [5]. To our knowledge, anti‐IL‐17 therapy is yet to be clinically evaluated in CRS patients. Despite recent advances, CRS endotyping still remains a challenge, owing to the lack of conclusively stratifying and specific biomarkers, illustrated by non‐uniform endotyping categories used in various publications. The matter is further complicated by patients with mixed endotypes. Real life data further shows that although dupilumab is generally a highly effective and well tolerated treatment option for patients with type II CRSwNP, the treatment effect is variable [6, 7]. We hypothesize that the occasional treatment failure with dupilumab seen in some patients could at least in some cases be the result of incorrectly endotyping those patients as type II. Since all the approved therapeutic antibodies for CRS target type II inflammation there is an unmet need for difficult‐to‐treat non‐type II and mixed endotype CRS cases.

## 6 Conclusion

In summary, we identified two patients with mixed Th2/Th17 endotype CRSwNP who showed favorable treatment outcomes with secukinumab treatment. Although larger‐scale studies are necessary, IL‐17 could be a promising target for mixed endotype or non‐type II CRSwNP. The current study further underscores the need for adequate biomarkers to facilitate a precision medicine approach in CRS.

## Author Contributions

**Mihkel Plaas:** conceptualization, data curation, investigation, methodology, project administration, resources, writing – original draft, writing – review and editing. **Agnieska Brazovskaja:** conceptualization, data curation, investigation, methodology, writing – original draft, writing – review and editing. **Liina Tserel:** conceptualization, methodology, writing – original draft. **Kai Kisand:** conceptualization, methodology, writing – original draft. **Pärt Peterson:** conceptualization, methodology, project administration, supervision, writing – original draft, writing – review and editing.

## Ethics Statement

The study was approved by the ethics committee of Estonian State Agency of Medicines and was conducted in accordance with the Good Clinical Practice Guideline. Informed consent was obtained from all participants.

## Consent

Written informed consent was obtained from all participants to publish this report in accordance with the journal's patients consents policy and Good Clinical Practice guidelines.

## Conflicts of Interest

Mihkel Plaas has received speaker fees from Sanofi and Astra Zeneca.

## Data Availability

The data that support the findings of this study are available from the corresponding author [MP], upon reasonable request.
